# *De Novo* Genome Assembly Shows Genome Wide Similarity between *Trypanosoma brucei brucei* and *Trypanosoma brucei rhodesiense*

**DOI:** 10.1371/journal.pone.0147660

**Published:** 2016-02-24

**Authors:** Mark Sistrom, Benjamin Evans, Joshua Benoit, Oliver Balmer, Serap Aksoy, Adalgisa Caccone

**Affiliations:** 1 School of Natural Sciences, University of California, Merced, 5200 N. Lake Rd, Merced, CA, 95343, United States of America; 2 Department of Ecology and Evolutionary Biology, Yale University, 21 Sachem Street New Haven, CT 06520, United States of America; 3 Department of Epidemiology of Microbial Diseases, Yale School of Public Health, New Haven, CT 06520, United States of America; 4 Swiss Tropical and Public Health Institute, Socinstrasse 57, 4051 Basel, Switzerland; University of Leicester, UNITED KINGDOM

## Abstract

**Background:**

*Trypanosoma brucei* is a eukaryotic pathogen which causes African trypanosomiasis. It is notable for its variant surface glycoprotein (VSG) coat, which undergoes antigenic variation enabled by a large suite of VSG pseudogenes, allowing for persistent evasion of host adaptive immunity. While *Trypanosoma brucei rhodesiense* (*Tbr*) and *T*. *b gambiense* (*Tbg*) are human infective, related *T*. *b*. *brucei* (*Tbb*) is cleared by human sera. A single gene, the *Serum Resistance Associated* (*SRA*) gene, confers *Tbr* its human infectivity phenotype. Potential genetic recombination of this gene between *Tbr* and non-human infective *Tbb* strains has significant epidemiological consequences for Human African Trypanosomiasis outbreaks.

**Results:**

Using long and short read whole genome sequencing, we generated a hybrid *de novo* assembly of a *Tbr* strain, producing 4,210 scaffolds totaling approximately 38.8 megabases, which comprise a significant proportion of the *Tbr* genome, and thus represents a valuable tool for a comparative genomics analyses among human and non-human infective *T*. *brucei* and future complete genome assembly. We detected 5,970 putative genes, of which two, an alcohol oxidoreductase and a pentatricopeptide repeat-containing protein, were members of gene families common to all *T*. *brucei* subspecies, but variants specific to the *Tbr* strain sequenced in this study. Our findings confirmed the extremely high level of genomic similarity between the two parasite subspecies found in other studies.

**Conclusions:**

We confirm at the whole genome level high similarity between the two *Tbb* and *Tbr* strains studied. The discovery of extremely minor genomic differentiation between *Tbb* and *Tbr* suggests that the transference of the SRA gene via genetic recombination could potentially result in novel human infective strains, thus all genetic backgrounds of *T*. *brucei* should be considered potentially human infective in regions where *Tbr* is prevalent.

## Introduction

African trypanosomiasis is a disease of humans and livestock in sub-Saharan Africa caused by protozoan parasites of the *Trypanosoma brucei* complex, which are transmitted between mammalian hosts by their tsetse fly (*Glossina sp*.) vector [1]. Human-infective members of the *Trypanosoma brucei* complex are the causative agents of Human African Trypanosomiasis (HAT), or sleeping sickness [2]. *T*. *b*. *rhodesiense* (*Tbr*) causes an acute form of HAT in eastern Africa, characterized by punctuated outbreaks in discrete disease foci [3], while *T*. *b*. *gambiense* (*Tbg*) causes a chronic form of the disease in western and central Africa and accounts for over 95% of reported cases [4]. *T*. *b*. *brucei* (*Tbb*), is not infective to humans, but, together with other animal trypanosome species, causes the livestock wasting disease, Nagana, across a range that overlaps with that of the human-infective parasites [2]. According to recent estimates from the World Health Organization, 50 million people in Africa are at risk of acquiring sleeping sickness. Although the number of new HAT cases has recently fallen below 10,000 for the first time in decades, the disease has a long history of cyclical emergence patterns [5]. This, coupled with the lack of a vaccine against HAT and high toxicity of late stage drug treatments [6], poses a significant challenge to the proposed goal of eliminating HAT as a public health problem by 2020 [7].

Two complete genome assemblies exist for one strain each of two of the three subspecies within the *T*. *brucei* group, *Tbb* [8] and *Tbg* [9]. A comparison of these genomes has revealed that, despite the substantial difference in disease caused by them, they are very similar at a genomic level—with 99.2% of sequence identity in coding regions and only a single oxidoreductase gene present in *Tbb* but not in *Tbg* [9]. Population level genomic comparison of 39 isolates sampled across the three named subspecies within the *T*. *brucei* group (*Tbb*, *Tbr* and *Tbg*) confirms a high degree of similarity, with only 2.33% of nucleotides being variable across the group, and no fixed SNP differences between them [10]. This genome wide analysis also confirms previous microsatellite data [11–15] that suggested that, while *Tbg* strains are genetically distinct from *Tbb*/*Tbr*, these strains are indistinguishable from one another [10]. Additionally, shared heterozygosity between a *Tbb* and a *Tbr* strain at the genomic scale [16] strongly suggests that horizontal transfer between the two subspecies occurs in the field. Conversely, a study of 7 microsatellite loci did not find evidence of gene flow between *Tbb* and *Tbr* [17]. This finding is in contradiction with numerous other population level studies that show that sympatric strains of *Tbb* and *Tbr* are more closely related to each other than to allopatric strains from the same named taxon [11–15]. This apparent contradiction is potentially due to the use of a small number of makers characterized by low diversity leading to limited ability to detect gene flow, rather than genuine reproductive isolation.

The ability of *T*. *brucei* to evade mammalian host adaptive immune response is through remarkable antigenic variation of its VSG coat, enabled by a suite of non-expressed VSG genes largely located in subtelomeric cassettes and on a variable number of small to intermediate sized chromosomes [8]. It is thought that replacement of expressed VSG genes with novel copies through ectopic recombination allows for the expression of a novel protein coat approximately once every 100 cell doublings during clonal replication [18]. ESAGs are co-transcribed with VSG genes, and pseudogenic copies are prevalent in sub-telomeric VSG arrays [19]. While the function of ESAG3 is not explicitly known, ESAGs are involved in recombination driven antigenic variation [20].

Despite high relative variability in the VSG and ESAG regions of the *T*. *brucei* genome, the number of genomic differences between subspeciess is remarkably low [9,21]. However, there are critical functional differences between *T*. *brucei* subspecies—specifically the ability of the human infective forms to evade human innate resistance via the action of the trypanosome lytic factors (TLF) present in the serum. Both *Tbg* and *Tbr* have independently evolved distinct mechanisms to evade the human immune system. *Tbg* evades lysis by TLF through a modified haptoglobin-haemoglobin (HpHbr) receptor and through the presence of a specific, truncated VSG (*TgsGP*) [22,23] that allows for reduced uptake and efficacy of TLF [19,24]. In *Tbr*, another truncated VSG (*SRA*) prevents cell lysis by binding to the TLF protein apolipoprotein L-1 (ApoL-1), the trypanolytic component of TLF [3,25]. Heterogeneous expression of SRA in previously susceptible *Tbb* strains renders them resistant to lysis by human serum [25]. Interestingly, no other genetic differences between the two subspecies are known and in laboratory tests *Tbr* and *Tbb* can sexually recombine in the tsetse fly vector to produce viable, recombinant offspring [26–28]. The possibility that the SRA gene is the only differentiating feature between *Tbb* and *Tbr* subspecies indicates that, if recombination occurs in wild populations of *T*. *brucei*, *Tbb* strains which are currently un-infective in humans, could potentially acquire SRA via genetic recombination, thus becoming infective [4,28]. This has significant epidemiological implications for at least two reasons: (1) As Rhodesian HAT is characterized by temporally and geographically localized outbreak foci [3], they may arise from recombination with previously un-infective *Tbb* strains, and thus not necessarily require *Tbr* movement between disease foci; (2) all *Tbb* genetic backgrounds must be considered potentially infective, when trying to predict and control outbreaks of Rhodesian HAT.

Although assembled and annotated genomes of *Tbb* and *Tbg* exist [8,9], it is not currently known if the SRA gene is the only gene specific to *Tbr*, because a *Tbr* genome is not yet available. Moreover, previous genomic studies of isolates from all three subspecies were based on short-read Illumina data aligned against the published *Tbb* genome, thus impeding our ability to detect *Tbr* specific variants not found in the *Tbb* genome. In the current study we produced a *Tbr* hybrid *de novo* assembly taking advantage of long-read Pacific Biosciences and short-read, high throughput Illumina sequence data. We used these sequences to extract putative genes from the *Tbr* genome to compare with the existing *Tbb* and *Tbg* genomes [8,9] to determine if any are only found in the *Tbr* genome. If genes specific to *Tbr* other than SRA are present, it would suggest that functions other than that conferred by the SRA gene are involved in the life history and disease type differences associated with *Tbr*. If no unique genes other than SRA are discovered, SRA gene is likely to be solely responsible for the human infectivity of *Tbr*. This would further suggest that any *Tbb* strain from independent evolutionary backgrounds could become human infective upon acquiring the SRA gene through horizontal transfer events.

## Results and Discussion

### Sequencing and Assembly

Short read high throughput sequencing of the STIB900 *Tbr* strain produced 48,975,696 individual reads for an expected coverage based on the TREU 927/4 *Tbb* genome of approximately 122x. Long read high throughput sequencing produced a total of 570,319 sequences. Read length of the Pacific Biosciences long read sequencing ranged from 116–9,729 bp (S1 Fig). Hybrid *de novo* assembly of the data resulted in 4,210 individual scaffolds ranging in length from 1,256–243,494 bp (S1 Appendix). The total number of base pairs included in scaffolds was 38,771,836. The number of reads *vs* read length and number of scaffolds *vs* scaffold length is shown in S1 Fig.

Given that the shortest of the 11 megabase (mb) chromosomes of the *Tbb* nuclear genome is approximately 1.1mb in length [8], the hybrid *de novo* assembly method we implemented was unable to recover full-length sequences of these chromosomes. This is probably in part due to the fact that the *T*. *brucei* genome is known to be extremely repetitive [8,9], presenting significant challenges to current methodologies for genome assembly. However, a promising result from our data is that the total length of our scaffolds (38.8mb) is considerably longer than the annotated *Tbb* genome (30.2mb), suggesting that the scaffolds produced in this study comprise a significant proportion of the *Tbr* genome, and thus are a valuable tool for a future complete genome assembly. However, the highly repetitive nature of the VSG subtelomeric libraries, which can comprise up to 30% of the *T*. *brucei* genome [8], makes these regions inherently difficult to accurately assemble, meaning that additional curation and resequencing is likely to be necessary to accurately construct them.

### Detection of Novel Genes

**Fig 1:**
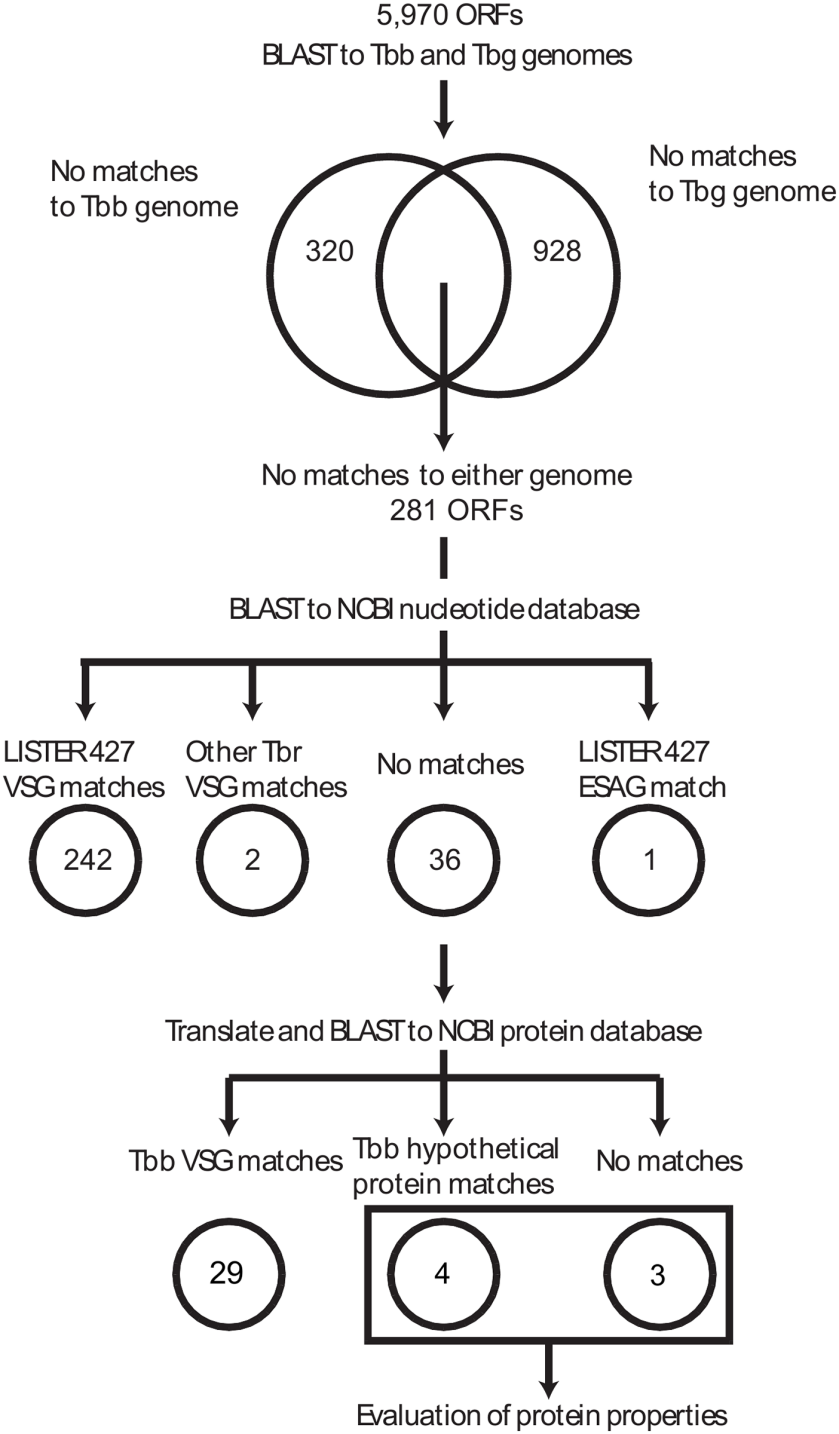
Flow chart documenting the series of BLAST searches and filtering leading from the initial 5,970 ORFs detected from the *de novo* assembly of the STIB900 *T*. *b*. *rhodesiense* genome to the final two genes specific to this *Tbr* strain. Of note is the high number of variant surface glycoprotein (VSG) genes, which show substantial specificity to the *T*. *b*. *rhodesiense* strain and were detected at each level of filtering.

To look for *Tbr* genes not found in the published *Tbb* or *Tbg* genomes we focused our analyses on the longest scaffolds (1,817 scaffolds >5000bp in length), which comprised 30.8mb of the *Tbr* genome. We detected a total of 5,970 open reading frames (ORFs) >1,000bp in length (S2 Appendix), which are likely to represent a significant portion of the *Tbr* genes, given that the number of genes in the *Tbb* genome is 9,898 [8]. In support of this we found that 85.9% of reads mapped to the *Tbb* genome [8] and that they were evenly distributed across the 11 chromosomes of the genome (S3 Appendix). The BLAST [29] searches and progressive filtering steps from the initial 5,970 ORFs to the final *Tbr* specific putative genes are summarized in Fig 1. We found 320 and 928 ORFs that did not have a match in the *Tbb* TREU 927/4 and *Tbg* DAL 972 genomes, respectively, confirming the higher level of similarity of *Tbr* with *Tbb* than *Tbg* found in previous studies [10–12]. Interestingly, we also found 281 ORFs in the *Tbr* strain we sequenced with no match to either the published *Tbb* or the *Tbg* genomes. When these were compared to the Genbank nucleotide database, 242 ORFs matched variant surface glycoprotein (VSG) pseudogenes from the well-studied LISTER 427 *Tbb* strain [30] and 2 ORFs matched VSG genes from clones from other *Tbr* strains. This complements significant efforts that have been made the characterize VSG variation across *T*. *brucei* [20,31] which has demonstrated that the majority of subspecies specificity in *T*. *brucei* lies in these gene regions, such that these 242 ORFs did not match to either reference genome, but did match to the LISTER 427 *Tbb* strain. It is also possible that despite painstaking efforts to characterize VSG cassettes in the T. brucei genome [20,31] the assembly of VSG cassettes in the annotated genomes of *Tbb* and *Tbg* are incomplete due to the inherent difficulties in assembling these highly repetitive genomic regions, thus not allowing for an exhaustive comparison of the three subspecies. We also found one ORF that matched to a LISTER 427 expression site associated gene (ESAG) pseudogene—ESAG3. Of the remaining 36 ORFs, which did not have a nucleotide match in the *Tbb* or *Tbg* genomes, 29 still matched to VSG genes, when translated into amino acid sequences and searched against the NCBI protein database. The accumulation of synonymous substitutions in these genes was probably sufficient to prevent a nucleotide, but not a protein match. This result implies functional conservation across the *Tbr* VSG library and also suggests a potential role for purifying selection operating on VSG arrays. Our analyses also revealed that over 96% of ORFs, which did not match to either the *Tbb* or *Tbr* genomes, were matches to VSG pseudogenes.

Four of the seven remaining ORFs (ORF 4–7, Table 1) have confident (>99% identity, 100% coverage) matches to hypothetical proteins present in the *Tbb* genome in the NCBI protein database. This implies that these genes are likely to be orthologous to coding genes in the *Tbb* genome, but with enough synonymous variation to prevent a confident nucleotide level match in either *Tbb* or *Tbg*. The remaining three ORFs (ORFs 1–3, Table 1) did not have matches in the NCBI protein database, and appear to represent putatively novel genes specific to the STIB900 *Tbr* genome. The first two of these three ORFs (ORFs 1 and 2, Table 1) are identical at the amino acid level, indicating multiple copies of this putative gene in the *Tbr* genome.

**Table 1:** Summary of statistics calculated, putative function and expression levels for genes of unknown function with no nucleotide level matches outside of the STIB900 *Tbr* genome. A) Statistics reported include length in nucleotides, Gravy Index (GI), Instability index (II), Isoelectric Point (IP), number of coiled coils (CC), longest disorder region in amino acids (DR), Percentage of coil structure (CS), number of trans-membrane helices (TH), number of signal peptides (SP), Insertion score (IS), number of homologs in the NCBI non-redundant protein database (HNr), number of homologs in the RCSB protein database (HPDB). B) Putative function is listed, as determined using a meta-prediction search and the method used to determine putative function.

A													B	
ORF	Length	GI	II	IE	CC	DR	CS	TH	SP	IS	HNr	HPDB	Putative function	Source
1	1749	-0.5	53	9	0	11	26	No	No	0	1012	40	pentatricopeptide repeat-containing protein	Hhsearch
2	1749	-0.5	53	9	0	11	26	No	No	0	1012	40	pentatricopeptide repeat-containing protein	HHsearch
3	1038	0.3	54	6	0	25	30	1	39	-	2	0	Alcohol Oxidoreductase	EzyPred
4	1452	-0.3	58	6	0	73	45	No	No	-	2	0	Hydrolase acting on ester bonds	EzyPred
5	2799	-0.3	53	8	0	35	51	No	No	0	15	0	Hydrolase acting on ester bonds	EzyPred
6	1251	0.2	28	8	0	54	35	10	No	0	810	0	Solute carrier family 35	Homolog in SWISS-PROT Database
7	1530	-0.6	61	6	21	115	47	No	No	-	1	0	None detected	

### Structure and Function of Novel Genes

We used the Xtalpred RF server [32] to investigate the biochemical and biophysical properties of the seven genes with unknown functions (ORFs 1–7). Table 1 shows for each of these ORFs the gravy and instability indices, isoelectric points, numbers of coiled coils, longest disorder region in amino acids, percentages of coil structure, numbers of trans-membrane helices, numbers of signal peptides, insertion scores, and numbers of homologs in the NCBI non-redundant protein database and in the RCSB protein database. We used Meta-Server for protein sequence analysis (MESSA) [33] to predict their function. As four ORFs have close homologs in the *Tbb* genome (ORFs 4–7, Table 1), we focused on the putative function of the remaining three ORFs for which no match in the *Tbr/Tbg* genomes was found. The results of this analysis suggest that the ORFs 1 and 2 encode a pentatricopeptide repeat-containing (PPR) protein, a family of genes critical in facilitating mitochondrial translation in trypanosomes [34], while ORF 3 encodes a putative alcohol oxidoreductase, an enzyme involved in alcohol metabolism in many organisms and implicated in drug resistance in *Trypanosoma cruzi* [35]. Of note is the fact that the single gene present in the published *Tbb* genome and absent from the *Tbg* genome is also an oxidoreductase gene [9]. This would suggest that while the conservation of oxidoreductase genes is characteristic of African trypanosomes [36], some specificity of function of oxidoreductase in certain strains may exist. It should be noted that translocation may explain the appearance of these seemingly novel gene variants—of note is observed high rates of translocation in PPR genes in plant genomes [37]. Possible translocation of these genes is warrants further analysis pending a more complete Tbr genome assembly.

**Fig 2:**
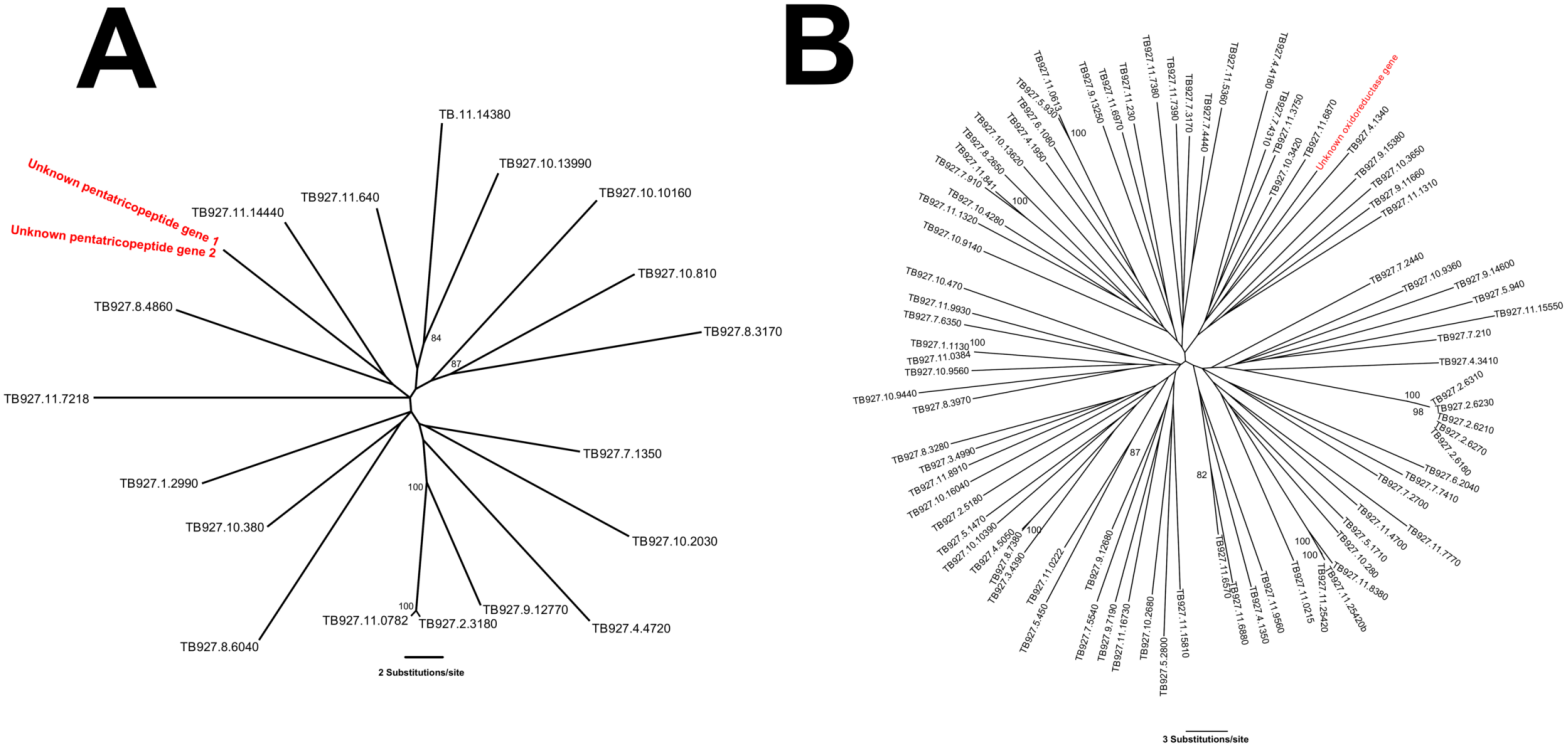
Phylogenies of A) ORF 1–2 with annotated pentatricopeptide repeat-containing protein genes in the TREU927/4 *Tbb* genome; and B) ORF3 with oxidoreductase genes in the TREU927/4 *Tbb* genome. These phylogenies confirm that these *Tbr* ORFs are related to members of these two gene families, but are phylogenetically distinct variants specific to the STIB900 *Tbr* strain. The unknown ORFs detected in our study are shown in red, known genes are shown in black, encoded using their TriTrypDB [36] database names. Bootstrap support values are shown for nodes with support >70.

We carried out phylogenetic analyses using RaxMLv7.7.6 [38] to: (1) clarify the evolutionary relationships between the ORFs 1–3 (Table 1) and their respective homologs in the *Tbr* genome, and (2) demonstrate that these ORFs are related to, but not identical to other PTP repeat containing and oxidoreductase proteins in the *Tbb and Tbg* genomes, respectively. Fig 2 shows the results for the two gene families. Both trees have low bootstrap support values on several nodes, limiting interpretations on the specific relationships among the different genes. This is likely due to relatively high levels of sequence divergence amongst genes causing phylogenetic saturation in the alignments. Nevertheless, regardless of the weakness of most topological relationships, this analysis clearly shows that ORF 1–3 are related *but not identical* to genes with similar functions in the *Tbb* genome. This further supports the possibility that these three ORFs, while members of the above mentioned gene families, may be *Tbr* specific variants. This possibility is also strengthened by considering the alignment of the flanking regions of each ORF to the *Tbb* and *Tbg* genomes. As the first two ORFs are located on a single scaffold, we aligned the regions of the scaffold before, between, and after the two ORFs. These regions all aligned to the first chromosome of the *Tbb* and *Tbg* genomes between 321,685–328,598bp, demonstrating that the flanking regions overlap with no ORF between them (S2 and S3 Figs, S4 Appendix). However, it is important to also note that this alignment is characterized by low pairwise identity (46.4%), suggesting that it may be misaligned. This could be due to the repetitive nature of the ORFs and flanking regions, which may be syntenic with a poorly assembled repetitive region of the *Tbb/Tbg* genome, or potentially the entire region is specific to *Tbr*. The region before and after ORF 3 aligned to chromosome 9 of the *Tbb* and *Tbg* genomes between 1,929,544–1,935,562bp. The flanking regions do not overlap, however the gap between them is 313bp—considerably shorter than the ORF (1,038bp), suggesting that the ORF is not present in either the *Tbb* or *Tbg* genome.

### Epidemiological and Evolutionary Implications

The *de novo* approach presented here provides the final proof of the genetic similarity between *Tbb* and *Tbr*, which was suggested by previous studies based on a microsatellite [11–15] and genomic comparisons [10,16]. The important implications of this result are at least twofold. First, considering *Tbb* and *Tbr* separate subspecies, although accepted in epidemiological practice, is misleading, because of the implicit assertion that taxonomic designation reflects independent evolutionary trajectories [28,39]. Second, this finding implies that all *Tbb* strains circulating in *T*. *brucei* non-human host have the potential to acquire the SRA gene and thus become human infective. Admittedly, given that recombination can only happen in the tsetse salivary glands, the likelihood of this happening frequently is relatively low, depending on how often tsetse flies are infected with both subspecies. However, since we have evidence of gene exchanges among sympatric *Tbb* and *Tbr* subspecies [11,15], this must have occurred over evolutionary times. Thus, this possibility and its epidemiological implications cannot be dismissed, as it suggests that epidemiological studies and control efforts would be significantly aided by a population scale analysis of the rate of gene flow between *Tbb* and *Tbr* subspecies in wild populations.

The investigation of almost 6,000 ORFs in the STIB900 *Tbr* strain reveals that only three 3 genes (ORF 1–3, Table 1) aside from the SRA gene are putatively specific to *Tbr*. This supports the previous suggestion that *Tbb*, *Tbg*, and *Tbr* are genetically highly similar [10–12,14] and that their observed differences in life history traits and disease outcomes are due to variation in genes present in all of them [9]. The fact that even the three genes (ORFS 1–3) found only in *Tbr* strain used in this study are members of gene families known to be abundant in trypanosomatids [9,34] further supports this point. Although these three ORFs seem to be *Tbr* specific, their role in directly facilitating human host infections is unclear, given that multiple copies of these genes are also found in *Tbb*. A similar pattern could also have been generated from convergent selection pressures due to similar selection regimes from exposure to the same host after independent strains of *Tbb* infected humans, following acquisition of the SRA gene. Moreover, as our analyses was based on the comparison of only one strain each for *Tbb*, *Tbg*, and *Tbr*, we cannot conclusively state that they are *Tbr* specific, as multiple strains for each subspecies from different geographic locations are necessary to test this. However, given the data we have so far, it remains plausible that acquisition of SRA is the only event required to allow a previously non-zoonotic *Tbb* strain to become human infective. Nevertheless, the finding of several VSG related ORFs and a few novel genes that appear to be *Tbr* specific suggest further research directions to better understand both their functional significance and evolutionary origin, as this may yield important insights for the development of novel treatments for Rhodesian HAT.

## Methods

### Sequencing and Assembly

We extracted DNA from a *Tbr* isolate (STIB900) from cryobanks at the Swiss Tropical and Public Health Institute, Basel. This strain was isolated from a patient in Ifakara, Tanzania in 1982 and had undergone minimal laboratory passaging. The presence of the SRA gene and thus as *Tbr* via PCR using the protocols outlined in [40]. Fragmentation and library preparation for both short and long read sequencing was carried out at the Yale Center for Genome Analysis (YCGA). Short read library preparation was conducted using an Illumina Paired-End DNA Sample Prep Kit (Illumina Inc., USA) and paired end (2x75bp) sequencing performed using the Illumina HiSeq 2000 platform. Quality control of reads was done using FastQC [41]. Long read library preparation was conducted using a Pacific Biosciences DNA template prep kit (Pacific Biosciences, USA), and 16 cells of Single Molecule, Real-Time (SMRT) sequence data were produced using a Pacific Biosciences *RS* II sequencer (YCGA).

We used the two-step PBcR (PacBio corrected reads) error correction and *de novo* assembly process described in Koren *et al*. [42]. This process, implemented in the Celera Assembler, trims and corrects individual long read sequences from Pacific Biosciences sequencing by mapping short read sequences from the Illumina platform to them to produce highly accurate, long read sequence for *de novo* assembly. *De novo* assembly was conducted using the Celera Assembler [43] using the default settings for long reads.

### Detection of open reading frames and blast search strategy

Scaffolds >5000 base pairs (bp) in length were imported into Geneiousv6.05 [35] to detect ORFs of at least 1000 bp—representing putative genes in the *Tbr* genome. ORFs were exported from Geneious and BLASTv2.27 [29] was used to detect matches to both the TREU 927/4 *Tbb* genome [8] and the DAL972 *Tbg* genome [9]. For all BLAST searches, we used an e-value of 1^−5^, a minimum length of 800bp, reporting only the best match for each ORF. Additionally, a BLAST search of the NCBI nucleotide database was conducted for ORFs for which no matches were found in either the *Tbb* or *Tbg* genomes. This was followed by a BLAST search against the NCBI protein database of the ORFs with no nucleotide match after translating then into amino acid sequences to identify potential functional matches. In order to confirm representative coverage of the genome, we aligned the detected ORFs to the *Tbb* genome [8] using BWAv0.7.12 [44]. Coverage for each chromosome was evaluated using Geneiousv6.05 [45] and detailed results for each chromosome reported in S3 Appendix.

### Determination of function and homology of novel genes

To understand more about three ORFs for which no confident match could be found (ORFs 1–3) and the four which matched genes of unknown function in the *Tbb* genome (ORF 4–7), we analyzed them with the program Xtalpred [32]. Xtlapred uses a logarithmic opinion pool method to determine the feasibility of a given protein to crystallize and estimates a number of parameters relevant to the secondary structure of the protein. We used the Meta Server for Sequence Analysis (MESSA) [33] to predict the putative function of each ORF. This method implements a variety of search strategies to predict the structure and function of a protein from its amino acid sequence.

To further examine genes with no close match in either the *Tbb* or *Tbg* genome, we extracted all genes annotated with the same predicted function (i.e. pentatricopeptide containing protein genes (ORF 1–2) (n = 20) and oxidoreductase genes (ORF 3) (n = 89) from the TREU 927/4 *Tbb* genome [8] and performed two separate alignments with the ORFs detected in our study using MUSCLEv3.8.31 [46]. Partitioning scheme and substitution model selection for each alignment was conducted using PartitionFinder v1.1.1 [47] which identified a model with 3^rd^ codon positions as a separate partition as optimal for both alignments and at GTR + I model as optimal for both 1^st^ and 2^nd^ codon partitions, and GTR and GTR+I+G models as optimal for the 3^rd^ positions of the ORF 1 and 2, and ORF 3 alignments respectively. A maximum likelihood phylogeny was constructed for each of the two alignments using RaxMLv7.7.6 [38] with 1000 bootstrap replicates. The consensus trees was visualized using Figtreev1.3.1 [48].

Additionally, we aligned the flanking regions of each of the genes with no close match in in either the *Tbb* or *Tbg* genome to those respective genomes to verify the absence of each ORF. Each alignment was conducted with MUSCLEv3.8.31 [46] and are shown in S4 Appendix.

## Ethics Statement

The isolate used for this study (STIB900) was collected by Dr Mantel Tanner as part of a diagnostic procedure in adherence to the medical ethics and the procedures of the Helsinki declaration for routine medical procedures.

## Supporting Information

S1 Appendix All scaffolds >5,000bp generated from the STIB900 *Tbr* genome using hybrid *de novo* assembly in multi-fasta format. (FASTA)

S2 Appendix The 5,970 ORFs detected in the STIB900 *Tbr* genome following hybrid *de novo* assembly in multi-fasta format. (CSV)

S3 Appendix Details of the alignment of the 5,970 ORFs to the Tbb genome, including the number of reads which mapped to each chromosome, the average coverage of each chromosome and maximum read depth found for each chromosome. (XLSX)

S4 Appendix Nexus alignment of the flanking regions of ORFs 1–3 to the *Tbb* and *Tbg* genomes in nexus format. A) The region before, between and after ORF 1 and 2 aligned with the first chromosome of the *Tbb* and *Tbg* genomes B) The region before and after ORF 3 aligned with the ninth chromosome of the *Tbb* and *Tbg* genomes.
(NEX)

S1 Fig A) The number Raw PacBio SMRT sequencing reads recovered plotted against read length in base pairs B) All scaffolds resulting from hybrid *de novo* assembly of combined PacBio SMRT read and Illumina short read data plotted against scaffold length in base pairs.
(PDF)

S2 Fig The best supported alignment of the flanking regions on the scaffold on which ORF1 and 2 were found before, between and after the two ORFs with the *Tbb* and *Tbg* genomes. The first three rows show the *Tbr* sequence before (first row), between (second row), and after (third row), compared to the sequence for chromosome 1 for *Tbb* (fourth row) and *Tbg* (fifth row). Colors indicate nucleotides (A—red, C—blue, G—orange, T—green). Dashed black lines represent putative indels. Numbers indicate genome positions in the *Tbb* and *Tbg* genomes and respective alignment positions in the flanking sequences.
(PDF)

S3 Fig The best supported alignment of the flanking regions on the scaffold on which ORF3 was found before and after the ORF with the *Tbb* and *Tbg* genomes. The first three rows show the *Tbr* sequence before (first row), between (second row), and after (third row), compared to the sequence for chromosome 9 for *Tbb* (fourth row) and *Tbg* (fifth row). Colors indicate nucleotides (A—red, C—blue, G—orange, T—green). Dashed black lines represent putative indels. Numbers indicate respective positions in the *Tbb* and *Tbg* genomes and respective alignment positions in the flanking sequences.
(PDF)
