# Supplementary figures and images for: De Novo Genome Assembly Shows Genome Wide Similarity between Trypanosoma brucei brucei and Trypanosoma brucei rhodesiense
Source: PLoS One. 2016 Feb 24;11(2):e0147660. doi: 10.1371/journal.pone.0147660 (PMC4766357; doi:10.1371/journal.pone.0147660)

**A**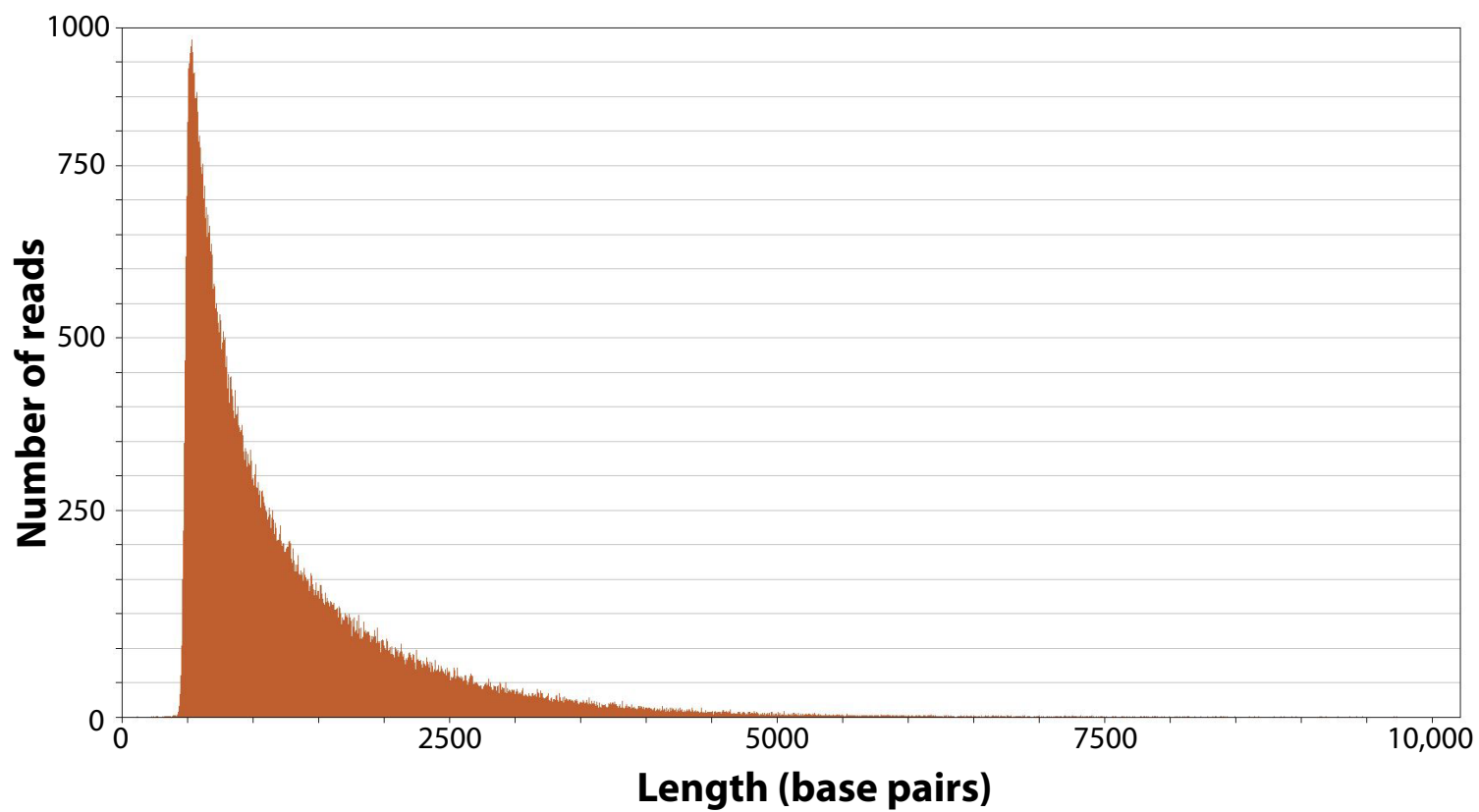**B**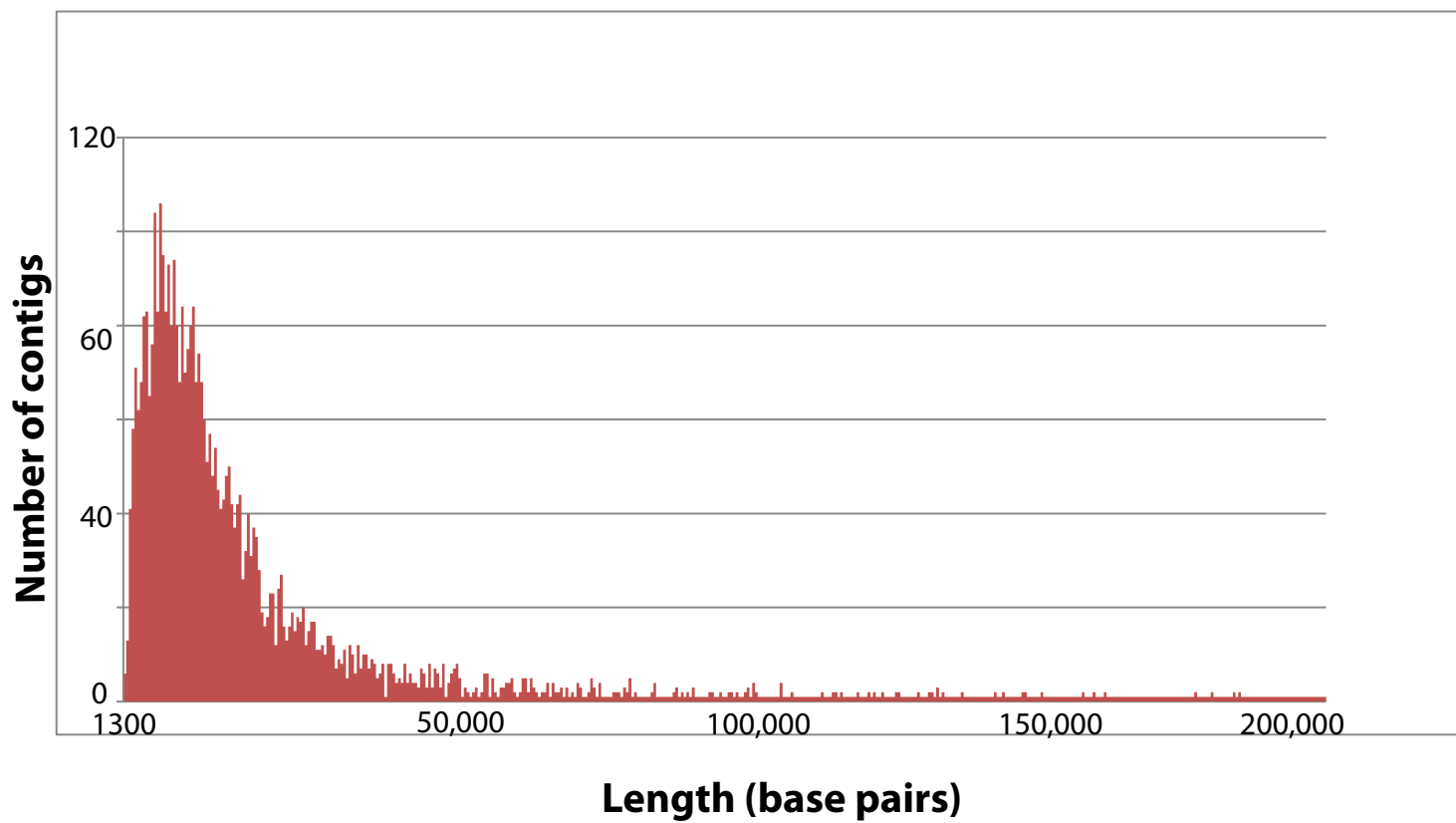

Supplement: S1 Fig — A) The number Raw PacBio SMRT sequencing reads recovered plotted against read length in base pairs B) All scaffolds resulting from hybrid de novo assembly of combined PacBio SMRT read and Illumina short read data plotted against scaffold length in base pairs. (PDF) [file pone.0147660.s005.pdf]

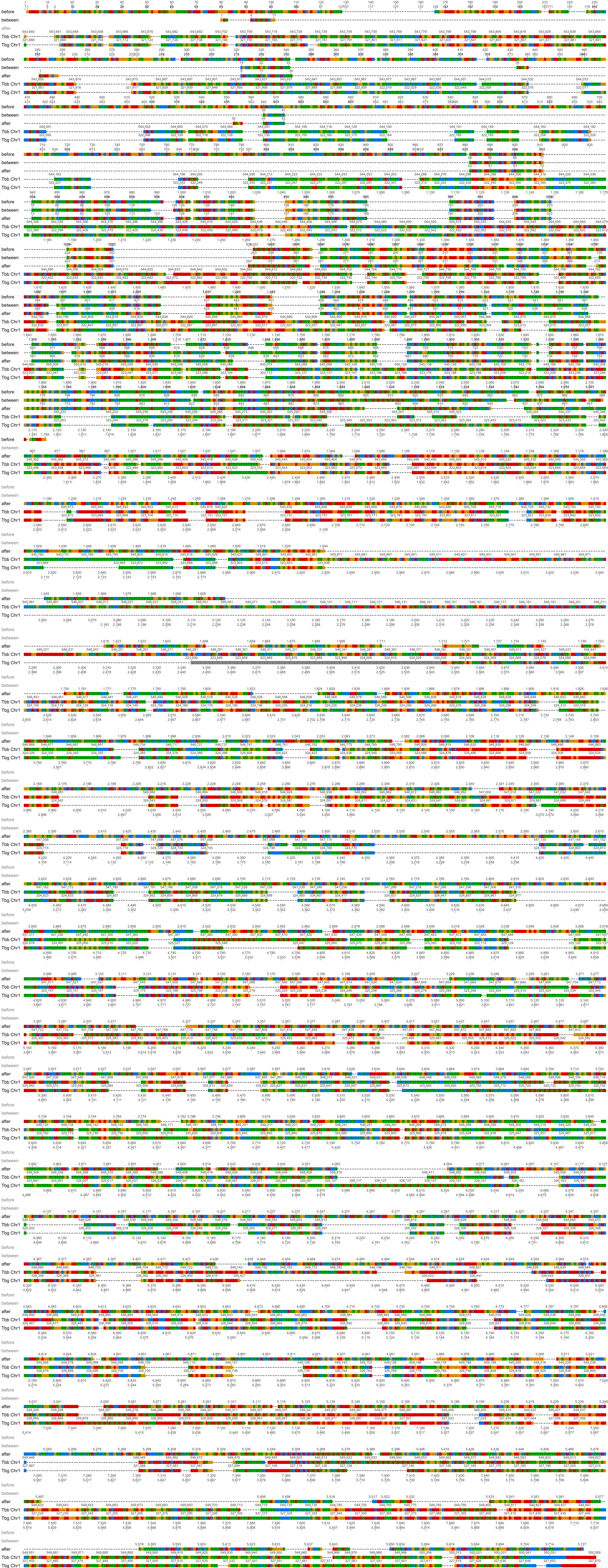

Supplement: S2 Fig — The first three rows show the Tbr sequence before (first row), between (second row), and after (third row), compared to the sequence for chromosome 1 for Tbb (fourth row) and Tbg (fifth row). Colors indicate nucleotides (A—red, C—blue, G—orange, T—green). Dashed black lines represent putative indels. Numbers indicate genome positions in the Tbb and Tbg genomes and respective alignment positions in the flanking sequences. (PDF) [file pone.0147660.s006.pdf]

before

after

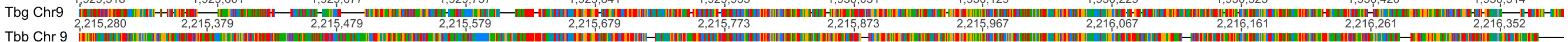

before

after

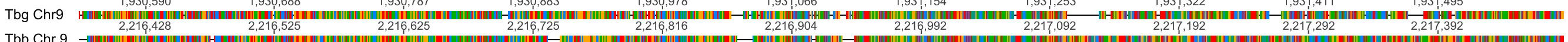

before

after

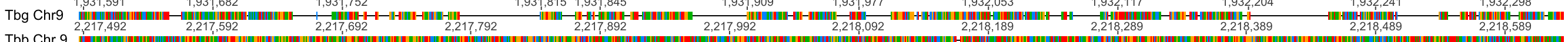

before

after

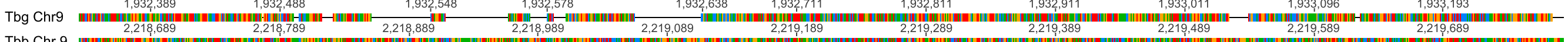

before

after

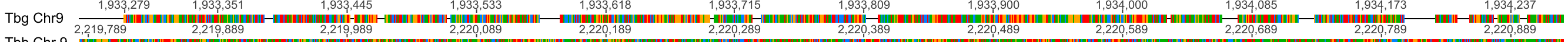

before

after

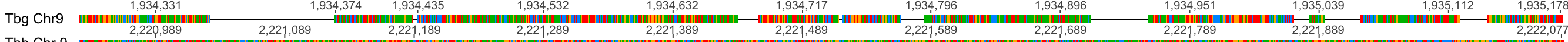

Supplement: S3 Fig — The first three rows show the Tbr sequence before (first row), between (second row), and after (third row), compared to the sequence for chromosome 9 for Tbb (fourth row) and Tbg (fifth row). Colors indicate nucleotides (A—red, C—blue, G—orange, T—green). Dashed black lines represent putative indels. Numbers indicate respective positions in the Tbb and Tbg genomes and respective alignment positions in the flanking sequences. (PDF) [file pone.0147660.s007.pdf]
